# Stochastic Decision Fusion of Convolutional Neural Networks for Tomato Ripeness Detection in Agricultural Sorting Systems

**DOI:** 10.3390/s21030917

**Published:** 2021-01-29

**Authors:** KwangEun Ko, Inhoon Jang, Jeong Hee Choi, Jeong Ho Lim, Da Uhm Lee

**Affiliations:** 1 Korea Institute of Industrial Technology, 143 Hanggaulro, Sangnok-gu, Ansan-si 15588, Gyeonggi-do, Korea; kke0217@kitech.re.kr; 2 Korea Food Research Institute, 245, Nongsaengmyeong-ro, Iseo-myeon, Wanju-Gun 55365, Jeollabuk-do, Korea; choijh@kfri.re.kr (J.H.C.); jhlim@kfri.re.kr (J.H.L.); dulee@kfri.re.kr (D.U.L.)

**Keywords:** tomato ripeness detection, convolutional neural networks, stochastic decision fusion, deep learning, automatic sorting system

## Abstract

Advances in machine learning and artificial intelligence have led to many promising solutions for challenging issues in agriculture. One of the remaining challenges is to develop practical applications, such as an automatic sorting system for after-ripening crops such as tomatoes, according to ripeness stages in the post-harvesting process. This paper proposes a novel method for detecting tomato ripeness by utilizing multiple streams of convolutional neural network (ConvNet) and their stochastic decision fusion (SDF) methodology. We have named the overall pipeline as SDF-ConvNets. The SDF-ConvNets can correctly detect the tomato ripeness by following consecutive phases: (1) an initial tomato ripeness detection for multi-view images based on the deep learning model, and (2) stochastic decision fusion of those initial results to obtain the final classification result. To train and validate the proposed method, we built a large-scale image dataset collected from a total of 2712 tomato samples according to five continuous ripeness stages. Five-fold cross-validation was used for a reliable evaluation of the performance of the proposed method. The experimental results indicate that the average accuracy for detecting the five ripeness stages of tomato samples reached 96%. In addition, we found that the proposed decision fusion phase contributed to the improvement of the accuracy of the tomato ripeness detection.

## 1. Introduction

The quality of tomatoes depends on appearance (color, size, texture, etc.) and nutritional value (minerals, acidity, antioxidants, etc.). These properties are commonly related to ripeness [1]. As tomatoes ripen, glucose and fructose accumulate, and therefore, antioxidants (e.g., ascorbate, lycopene, β-carotene, rutin, and caffeic acid) increase [2,3], organic acids (e.g., malic acid and citric acid) decrease, and sweetness increases [4]. Furthermore, the surface color changes to red owing to a decrease in chlorophyll and an increase in lycopene, and the flesh firmness decreases owing to a decrease in pectic substances [4,5]. Therefore, determining the appropriate ripening stages of tomatoes for sale before packaging is very important. For example, let us suppose that tomatoes in different ripeness stages are packaged into the same bundle. The tomatoes have different respiration rates, thereby resulting in the acceleration of ripening due to ethylene production. This effect makes quality management a challenge. On the other hand, the commercial value of tomatoes can be maintained longer if they are sorted and packed into the proper ripening stages [6]. The classification of ripening stages is generally conducted by trained laborers. The manual sorting process follows standard guidelines, such as those prescribed by the United States Department of Agriculture (USDA) chart [7]. This manual-based approach has drawbacks, such as relying on the competence of laborers and adding additional costs for training sorters. The biggest challenge that the agricultural industry faces is a decrease in the number of laborers due to an aging population and the rise of labor costs. These problems show that time-consuming and labor-intensive work should be replaced with automated systems in future farm environments.

In recent years, artificial intelligence technologies have become popular in the food and agriculture research field. In several recent studies related to food and agricultural production, machine learning and deep learning applications have shown great success. In the food domain, deep learning has shown promising performance in a variety of tasks, such as food recognition [8], calorie estimation [9], sentiment analysis for cookery channels [10], and fruit quality detection. In this paper, we narrow this scope to the core task of developing smart technology for tomato farms: tomato ripeness detection consisting of the localization of the tomato sample and its ripening stage classification. Fast and accurate detection of ripe tomatoes is an important task in replacing manual laborers with automatic systems. Zhao et al. [11] developed a machine vision system to detect ripe tomato samples in a greenhouse scene by combining the AdaBoost classifier and a contour analysis method. Liu et al. [12] studied an algorithm combining a coarse-to-fine scanning method and a false-color removal method to detect mature tomatoes. To achieve accurate ripe tomato detection, Hu et al. [13] suggested a method that combines a deep learning algorithm and an edge-contour analysis method. Sun et al. [14] proposed an improved feature-pyramid-network-based tomato organ recognition method. These results demonstrate that machine learning, including deep learning, contributes to improving tomato detection and can be further used in commercial applications.

Previous related works on fruit defect or grade detection in computer vision can be categorized into two types: approaches based on hand-crafted features and those using deeply learned features. Most existing studies belong to the former class [11,12,15,16,17]. Hand-crafted features have the advantages of locality and simplicity, but may lack the semantic and discriminative capacity of extracted features in changing environments, as appropriate features are generally selected based on experience. For example, as the number of ripening stages of a tomato sample increases, the difficulty in designing proper descriptors sufficient to classify these classes using raw images also increases. Furthermore, in the case of classification, it is very time-consuming to determine an optimum combination of the feature extractor and classifiers. In contrast, a deeply learned feature is extracted from the training dataset itself using an end-to-end learning model architecture, so it comes up with a reasonable descriptor for ripe tomato detection. Besides, the time-consuming procedure necessary to find the optimum combination of feature extractor and classifier is not required. For instance, a convolution network has abstracted feature maps that vary depending on the depth of the corresponding layers, so that any feature map can enable the representation of a data-driven descriptor [18]. Kamilaris et al. [19] found that deep learning models achieved higher accuracy compared with those using hand-crafted and shallow approaches. The fine-grained ripeness classification in practical scenarios is another challenge. Previous research has mainly focused on hand-crafted color features on the surface of tomatoes. Li et al. [20] proposed a dominant color histogram matching method to analyze the shape, ripeness level, size, and surface defects of tomatoes. Arakeri et al. [21] developed a tomato sorting software combining a preprocessor for noise filtering of raw RGB images and a color feature extractor to detect surface defects and the ripeness stage of tomatoes. In recent years, researchers have developed machine-learning-based approaches to classify the ripeness of tomatoes. For example, Goel and Sehgal [15] converted color features in RGB space into R–G features and conducted a sorting task using a fuzzy-based classifier. El-Bendary et al. [16] determined the ripeness degree using color features in the HSI space, a PCA-based feature extractor, and supervised-learning-model-based classifiers. Furthermore, recent research has shown promising performance for the same task [17]. However, these approaches may lead to intra-class variations at the same stage, such as dynamic viewpoints, illumination conditions, and atypical shapes of surface color distribution. These attributes are similar to the problem of human action recognition in videos. The two-stream convolutional neural networks (ConvNets) outperformed in the task of action recognition [22]. This scheme can be adapted to our problem of tomato ripeness detection by observing a tomato from multiple viewpoints, rather than from a single viewpoint. However, the late fusion of ConvNet streams may lead to performance decay if the fusion strategy is inadequate or the proper parameter settings are omitted. To solve this issue, we propose a novel tomato ripeness detection pipeline based on multi-ConvNet streams with a stochastic decision fusion (SDF) that can not only precisely classify the ripening stage of a tomato sample but also localize the sample in real time.

This paper aims to develop an accurate tomato ripeness detection method based on deep learning, called stochastic decision fusion of convolutional neural networks (SDF-ConvNets). The proposed method is expected to be applied in the form of a sorting system that classifies fruits according to those ripeness degrees in the post-harvest stage. Since the ripeness detection process is conducted by observing images from various viewpoints, excellent synergy can be expected if the sorting module can be designed based on a conveyor structure capable of rotating fruit objects and transporting them. To train and evaluate the model, we constructed a large-scale image dataset collected by our customized image acquisition system. Experiments for evaluating our system have shown superior performance to other methods. In summary, the key contributions of our work are: (a) developing a deep-learning-based robust and accurate ripe tomato detector, (b) increasing the accuracy of ripening stage classification using the stochastic decision fusion method, and (c) collecting a large-scale image dataset that captures the five ripening stages of tomatoes from different viewpoints.

## 2. Materials and Methods

### 2.1. Tomato Image Acquisition

**Figure 1:**
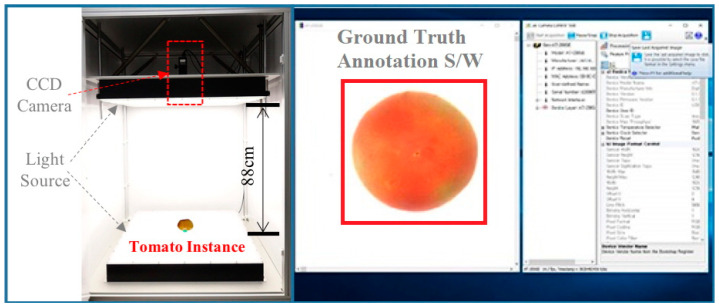
Camera system for acquisition of a large-scale image dataset of “Dafnis” variety tomatoes.

The tomato images used in this study were captured using a JAI 3CCD camera and a light source chamber, as shown in Figure 1. The CCD camera is a 3 × 1/3″ CCD color progressive scan camera (up to 120 frames/s with full resolution). The camera was coupled with a C-mount lens module whose focal length is 35 mm and the min/max operation range of the iris is f2.0/f22.0.

We designed the customized image acquisition system in conjunction with an annotation labeling software that can manually generate ground truth labels for training the proposed deep learning model at the same time as image acquisition. The ground truth labels consisted of the spatial information of tomatoes in the image space as well as the ripening stages. We selected tomatoes of the “Dafnis” variety for building a large-scale image dataset. These were classified into five stages according to the USDA color chart. The dataset was collected from a minimum of 500 samples for each ripeness stage to contain as many atypical features as possible, as shown in Table 1.

### 2.2. Accurate Tomato Ripeness Detection Using the SDF-ConvNets

We concentrated on an approach for classifying the ripening stage of tomatoes, considering practical scenarios such as an automated sorting application in the post-harvesting process.

**Figure 2:**
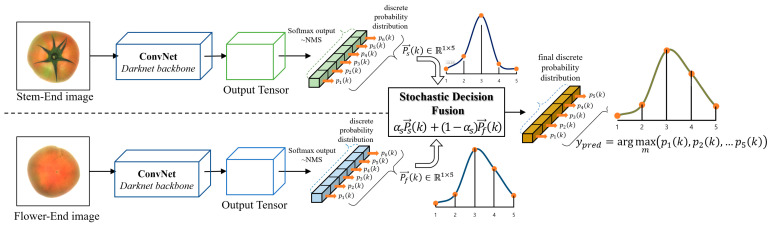
The workflow of stochastic decision fusion of convolutional neural networks (SDF-ConvNets) for tomato ripeness detection (NMS: non-maximum suppression).

We built a sequential process consisting of an initial tomato ripeness detection phase and a stochastic decision fusion phase (Figure 2). A standard one-stage detector YOLOv3 [23] was used for the initial ripeness detection stage based on tomato images viewed at the stem-/flower-end, respectively. Then, the estimated results were transferred to the stochastic decision fusion phase to improve the final ripening stage classification result of the target tomato sample. This approach was inspired by how human laborers generally judge the ripening stage of tomatoes by observing them from multiple viewpoints.

#### 2.2.1. Initial Tomato Ripeness Detection Based on YOLOv3

The initial tomato ripeness detection task consisted of two main parts: localizing the spatial region of the target tomato sample and classifying those ripening stages from tomato images viewed from the stem-/flower-end. Even when the observed environment is constrained, classifying ripening stages is a difficult fine-grained problem, in which the variation between consecutive stages is low and the variation between tomatoes belonging to the same group is relatively high. Recently, deep-learning-based approaches have been used as a solution to this type of problem. The typical ConvNet architecture consists of two main parts: a set of convolutional layers that perform feature extraction, and classification layers. The frontal layers of the network mainly focus on obtaining deeper domain features of the input, and the extracted features are transferred to the classification layers to discriminate between classes, such as the ripening stages by using fully connected [24] or global average pooling layers [25]. The parameters of the convolutional and classification layers can be trained end-to-end. Several studies on object classification and detection based on ConvNets have already achieved great success in various computer vision areas [26,27,28,29].

We applied a one-stage object detector based on YOLOv3 to resolve the tomato ripeness detection problem. The YOLOv3 starts with an assumption that the entire input image can be divided into *S* × *S* grid cells, and B proposal regions are located on each cell. The detector generates an output tensor consisting of five elements, such as the spatial information and class score for each region. If there is a jth bounding box in the ith grid cell, then the spatial information of the target object is depicted as, $b_{ij}=\left[b_{x},b_{y},b_{w},b_{h}\right]\in {\mathbb{R}}^{1\times 4}$ and the object class score for the bounding box is depicted as C. The spatial information is the coordinate offset and the size of the bounding box. The scalar variable C refers to whether the confidence score of the predicted box contains an object. Each box also predicts the multi-label score vector $P={\left[p\left(c_{m}\right)\right]}_{m=1,\ldots ,M}\in {\mathbb{R}}^{1\times M}$ as a result of independent logistic classifiers. The vector represents the conditional probability distribution of M classes, given that an object is contained in the predicted box. Therefore, the total size of the output tensor can be computed as S×S×B×(5+M). A post-processing step, such as a non-maximum suppression algorithm, is required to obtain the final result based on the output tensor. In the training phase, a loss function based on binary cross-entropy was applied. The loss function L consisted of sub-loss functions $L_{bbox}$, $L_{conf}$, and $L_{cls}$. First, $L_{bbox}$ was obtained by comparing the predicted bounding box $b_{ij}$ with the ground truth box $\hat{b},$ as shown in Equation (1). If the jth bounding box contains an object in the ith grid cell, then $1_{ij}^{obj}=1$, otherwise 0. It should also be noted that $1_{ij}^{nobj}=1-1_{ij}^{obj}$. The function d(u,v) indicates the Euclidean distance between two vectors u and v.

(1) $$L_{bbox}=\lambda_{bbox}\overset{S^{2}}{\underset{i=1}{\sum }}\overset{B}{\underset{j=1}{\sum }}1_{ij}^{obj}\left[d\left(b_{ij},\hat{b}\right)\right]$$

$L_{conf}$ represents the difference between the predicted confidence score C and the ground truth $\hat{C}$ among {0, 1}, as defined in Equation (2).

(2) $$L_{conf}=\overset{S^{2}}{\underset{i=1}{\sum }}\overset{B}{\underset{j=1}{\sum }}1_{ij}^{obj}\left[d\left(C_{ij},\hat{C}\right)\right]+\lambda_{nobj}\overset{S^{2}}{\underset{i=1}{\sum }}\overset{B}{\underset{j=1}{\sum }}1_{ij}^{nobj}\left[\left(C_{ij},\hat{C}\right)\right]$$

Equation (3) defines the loss $L_{cls}$ used in the general multi-label classification problem. $y_{m}$ is the ground truth label, so that if the class is correct it is 1 and if not, 0. If the correct one is the mth class, then $y_{m}=1$ and otherwise it is 0.

(3) $$L_{cls}=-\frac{1}{M}\overset{S^{2}}{\underset{i=1}{\sum }}\overset{B}{\underset{j=1}{\sum }}1_{ij}^{obj}\overset{M}{\underset{m=1}{\sum }}y_{m}\log\left(p\left(c_{m}\right)\right)+\left(1-y_{m}\right)\log\left(1-p\left(c_{m}\right)\right)$$

Finally, the sub-loss functions are defined in Equation (4).

(4) $$L=L_{bbox}+L_{conf}+L_{cls}$$

The weights applied to losses (1) and (2) were set as $\lambda_{bbox}=5$ and $\lambda_{nobj}=0.1$, respectively [29]. By optimizing this loss function according to the mini-batch-based stochastic gradient descent algorithm, each ConvNet can be trained.

**Figure 3:**
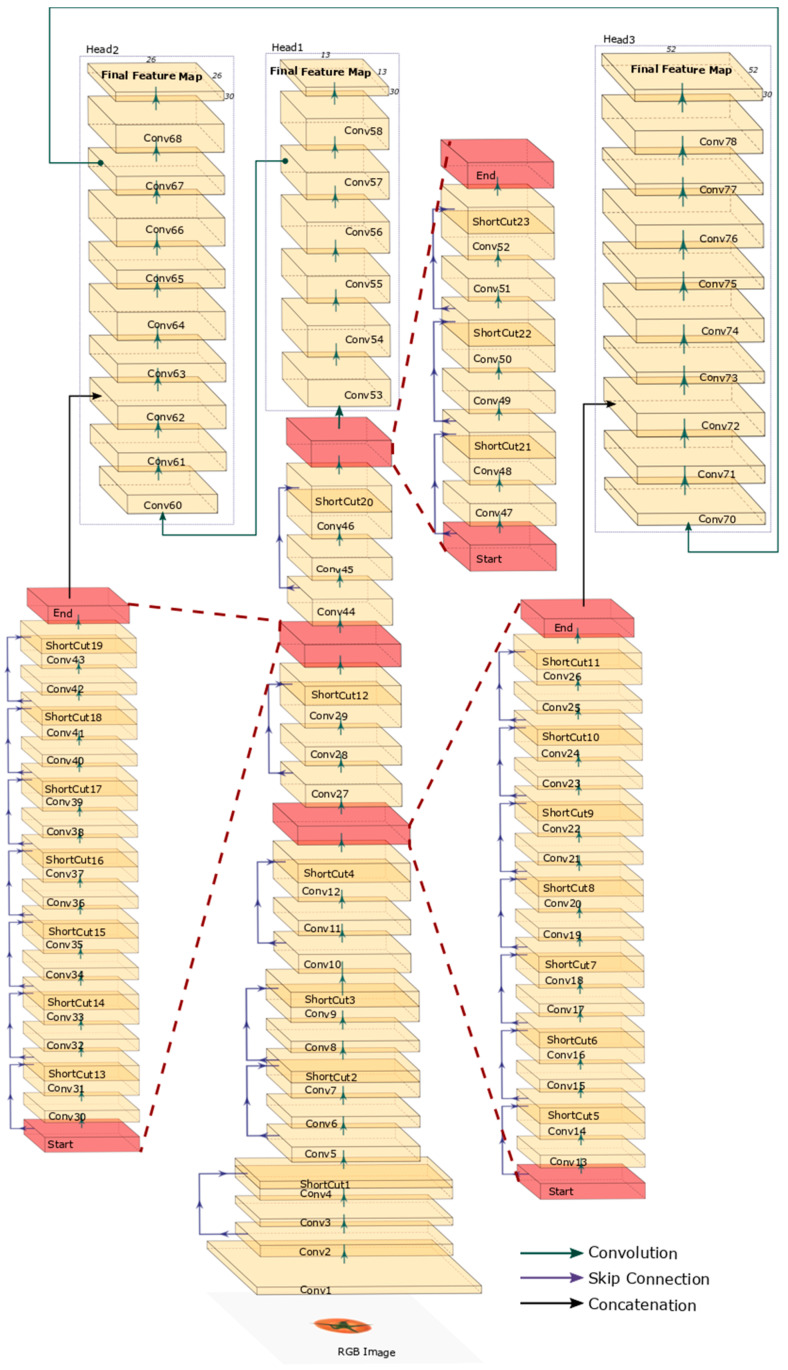
Visualization of backbone architecture for tomato ripeness detection.

We tried to arrange the backbone network architecture by piling multiple residual modules with a simple shortcut connection [27]. The ConvNet performance is strongly related to the hyperparameters of the backbone architecture. It is widely known that deeper models decrease bias and increase variance [30]. Considering the bias–variance tradeoff, we mainly focused on the number of convolution layers and scales of the final feature maps. There were 23 shortcut-connection-based residual modules and three heads conducting initial ripeness detection using the global average pooling layer, and each head received a different scaled feature map as input. The first head was directly derived from the backbone and utilize the smallest-scale feature map. The second head branched by using the low-level feature map of the backbone and the convolution layer output of the first head as input. The third head also branched by using the lowest-level feature map of the backbone and the convolution layer output of the second head. The detailed backbone ConvNet architecture of the ripeness detection phase is presented in Figure 3.

We trained the model with the hyperparameter configuration of the YOLOv3 [23]. The optimizer of the training process was a mini-batch stochastic gradient descent with momentum.

- Learning rate: 0.01 (scale 0.1 at step 25,000, 35,000);
- Max. training iteration: 50,000;
- Size of mini-batch/subdivision: 32/8;
- Weight decay: 0.0005;
- Learning momentum: 0.9;
- Total number of convolution layers: 79;
- Scales of final feature map: 8, 16, 32.

**Figure 4:**
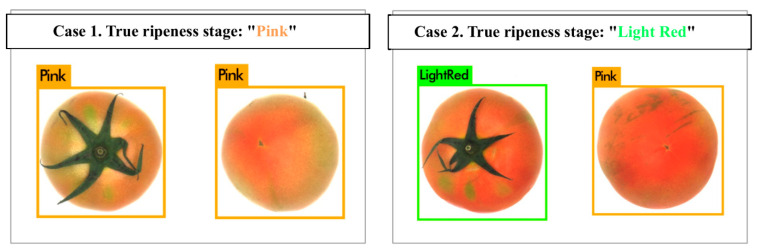
Examples of initial ripeness detection results of using ConvNet stream based on YOLOv3.

Figure 4 represents examples of the initial ripeness detection result based on the deep learning model. In case 1, the predicted stages at both viewpoints are matched, while the result at the flower-end viewpoint in case 2 is different from that at the other viewpoint. Therefore, in case 2, it is difficult to determine which is the correct ripening stage of the target tomato sample. To overcome this limitation, we propose a stochastic decision fusion method.

#### 2.2.2. Stochastic Decision Fusion

To accurately classify the final ripening stages of target tomato samples, we tried to apply two types of weighted-fusion-based approaches. The first was to assign equal weight to both ConvNet stream results. We assigned the equivalent scalar value as the weight for each stream, as shown in Equation (5):

(5) $$P=\overset{2}{\underset{n=1}{\sum }}0.5*P_{n}$$

where $P_{n}\in {\mathbb{R}}^{1\times M}$ is a multi-label score vector representing discrete probability distributions for tomato ripening stages when viewing a tomato sample from the n-th viewpoint.

Second, we hypothesized that weighting the superior one among the streams would increase the accuracy of the final decision. In this paper, a multi-label confusion matrix $A_{n}=⎣a_{n1},\ldots ,a_{nm},\;\ldots ,a_{nM}⎦\in {\mathbb{R}}^{M\times M}$ was used to reflect the performance value of each ConvNet stream in the weight decision process. The column vector $a_{nm}\in {\mathbb{R}}^{M\times 1}$ of the confusion matrix $A_{n}$ was normalized by the total number of samples belonging to the m-th class. This implies that each element of the $a_{nm}$ ratio of the number of samples classified as each class to the total number of samples belonging to the m-th class. The m-th element is regarded as the precision of the classification result. Precision is an appropriate performance metric for each ConvNet stream for classifying the ripening stages of tomatoes, as decreasing the number of false-positive samples is important for practical applications. Subsequently, the proposed weight decision process was conducted by combining the score vectors $P_{1},P_{2}$ and multi-label confusion matrices $A_{1},A_{2}$. The details are described based on examples of score vectors and confusion matrices. First, let us suppose that score vectors obtained from both ConvNet streams for the k-th tomato sample are set to $P_{1}\left(k\right)=\left[0.8,\;0.1,\;0.05,\;0.025,\;0.025\right]$ and $P_{2}\left(k\right)=\left[0.1,\;0.8,\;0.1,\;0,\;0\right]$, respectively. The multi-label confusion matrices for the results of both ConvNet streams are set to $A_{1}$ and $A_{2}$, as shown in Table 2.

For the element with $P_{1}\left(k\right),$ the largest score value belongs to the first ripening stage “T”, so the first column vector $a_{11}$ of $A_{1}$ is responsible for determining the weight of ConvNet stream 1. In contrast, the element of $P_{2}\left(k\right)$ belongs to the second stage “P” showing the largest score, so the second column vector $a_{22}$ of $A_{2}$ is responsible for determining the weight of ConvNet stream 2. Therefore, new weight vectors $\alpha_{1},\;\alpha_{2}\in {\mathbb{R}}^{1\times M}$ were computed using Equation (6).

(6) $$\alpha_{1}=\frac{a_{11}}{a_{11}+a_{21}},\;\alpha_{2}=\frac{a_{22}}{a_{12}+a_{22}}$$

**Figure 5:**
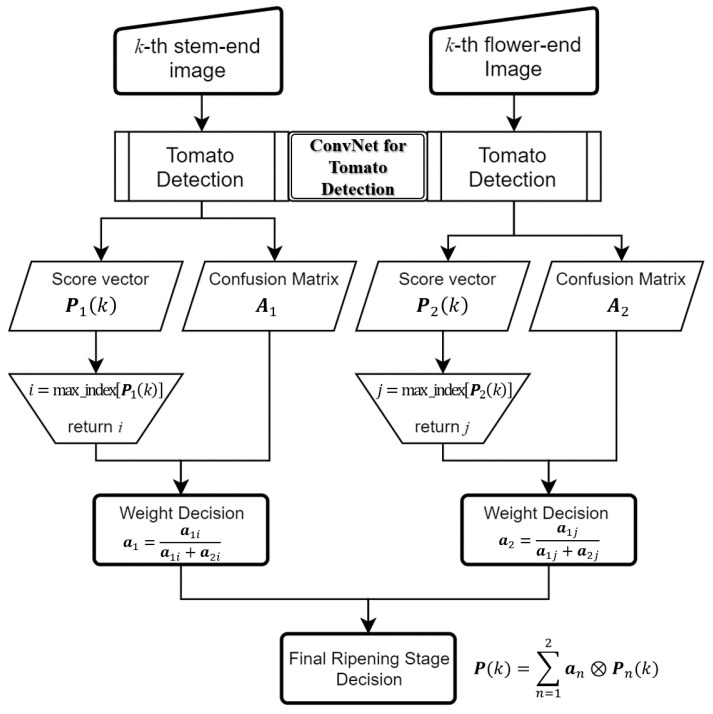
Flowchart of the proposed stochastic decision fusion algorithm.

The final ripening stage of the input tomato sample was computed as shown in Equation (7):

(7) $$P=\overset{2}{\underset{n=1}{\sum }}\alpha_{n}⊗P_{n}$$

where ⊗ is element-wise multiplication. These are given by the classification results of both ConvNet streams. Therefore, the final result depends on the configuration of the weight vectors, $\alpha_{1},\alpha_{2}\in {\mathbb{R}}^{M\times 1}$ which are, respectively, responsible for the stem-end and flower-end viewpoints of the tomato. This approach improves the accuracy of the ripening stage classification result by biasing the superior stream. The proposed algorithm is summarized in Figure 5. The results of both decision fusion processes are transformed into an L2-normalized vector like the Softmax function. Therefore, it is possible to determine the final ripening stage of the target tomato sample with the index of the maximum valued element of the output vector. In the next chapter, we describe several experiments we conducted to evaluate our proposed approach by comparing it with existing state-of-the-art approaches.

## 3. Results

The proposed SDF-ConvNets was verified with our tomato image dataset through experiments in this section. The experiments were conducted on a computer equipped with an Intel^®^ Core™ i7-4790K 4.00 GHz CPU, 32 GB of RAM, and an NVIDIA GeForce GTX Titan Xp GPU processor. We utilized the deep learning framework Darknet [31]. Three metrics were used to evaluate the experimental results: precision, recall, and F1 score. In the multi-class classification problem, we calculated the precision, recall, and F1-score per class in a one-versus-rest manner.

(8) $$Precision\left(class=c\right)=\frac{TP\left(c\right)}{TP\left(c\right)+FP\left(c\right)},\;Recall\left(c\right)=\frac{TP\left(c\right)}{TP\left(c\right)+FN\left(c\right)},$$

where TP/FP/FN is the number of true-positive/false-positive/false-negative samples of class *c*. Then, the per-class F1-score can be computed by Equation (9).

(9) $$F1\left(class=c\right)=\frac{2\times Precision\left(c\right)\times Recall\left(c\right)}{Precision\left(c\right)+Recall\left(c\right)}$$

### 3.1. Experiments for Tomato Ripeness Detection

Table 3 represents the experimental results obtained with our test dataset. The average F1-scores of 94.2% and 93.04% were achieved by the single ConvNet stream with flower-/stem-end images, respectively. It seems that the single ConvNet-based tomato ripeness detector without any decision fusion steps performed well for our dataset.

### 3.2. Experiments for Stochastic Decision Fusion

In this paper, we used two decision fusion approaches based on stochastic metrics for the final decision on the tomato ripeness stage of the target sample. As a result of comparing the ripeness detection performance according to the proposed decision fusion method with the results in Table 3, it can be seen that the decision fusion strategies contributed to improving the ripeness detection accuracy, as shown in Table 4. In addition, the proposed stochastic decision fusion technique was superior to the simple method of assigning equal weights.

### 3.3. Comparison of State-of-the-Art Algorithms

**Figure 6:**
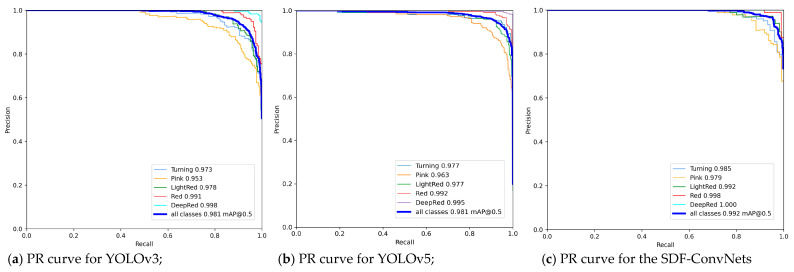
Precision–recall curves for three deep learning models for tomato ripeness detection with the same test dataset: (**a**) YOLOv3; (**b**) YOLOv5; (**c**) the SDF-ConvNets.

We also used the precision–recall (PR) curve to compare the SDF-ConvNets to other recent models, such as SVM [32] and YOLOv5 [33]. Three PR curve graphs are plotted in Figure 6. The first and second curves are the ripeness detection results using YOLOv3 and v5, respectively, and the last graph shows the SDF-ConvNets-based detection result. We can verify that the difference in performance between YOLOv3 and YOLOv5 was not noticeable, whereas the detection performance of the SDF-ConvNets was improved through the area under each PR curve.

These experimental results demonstrate that the proposed SDF-ConvNets outperformed other methods. Table 5 compares the recent achievement of related works for the fruit ripeness detection with the performance of the SDF-ConvNets to prove the result. We can see that our approach is superior given the number of classes that need to be classified or the number of images for testing.

## 4. Conclusions

In this paper, we proposed an accurate tomato ripeness detection methodology called SDF-ConvNets. The overall ripeness detection pipeline consisted of two major steps: the initial tomato ripeness detection phase based on ConvNet streams, and the stochastic decision fusion phase to obtain a more precise ripeness classification result. Even if the initial ripeness classification fails for the stem-end or flower-end tomato image, the proposed decision fusion phase can compensate for the misclassified stage into the correct stage. To train, test, and verify the proposed method, a large-scale image dataset was collected and labeled. The scale of the tomato image dataset is larger than any dataset used in recent related works. The dataset consisted of 2166 tomato samples for training ConvNets and 546 tomato samples used to evaluate the SDF-ConvNets. The experimental results were obtained by averaging 5-fold cross-validation and evaluated in terms of the three statistical metrics (precision, recall, and F1-score) of the tomato ripeness detection task. The SDF-ConvNets successfully achieved accurate and fine-grained tomato ripeness detection compared with other deep-learning-based approaches. The F1-score of the tomato ripeness detection using the SDF-ConvNets was 96.5%. The proposed method was compared with the recent achievement of related ripeness detection tasks and its superiority was demonstrated.

In future work, a follow-up study will be conducted to develop an integrated framework that can determine the appropriate harvest time and monitor the crop growth status by recognizing and estimating the ripening stage in real-time through the observation of tomatoes before harvest.

## Figures and Tables

**Table 1:** The number of tomato samples used for model training and ripeness detection sets.

Ripening Stages		Turning (T)	Pink (P)	Light Red (L)	Red (R)	Deep Red (D)
Sample Image (Dafnis)	Flower-end viewpoint	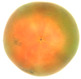	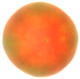	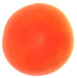	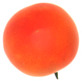	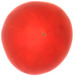
	Stem-end viewpoint	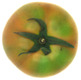	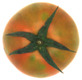	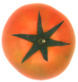	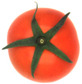	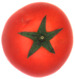
Number of Samples for Training Set		416	417	458	429	444
Number of Samples for Test Set		105	114	92	119	118

**Table 2:** Example of multi-label confusion matrices for ConvNet streams with stem-end view input (left) and flower-end view input (right). (T: Turning, P: Pink, L: Light Red, R: Red, D: Deep-Red)

Stem-End View Image		Prediction					Flower-End View Image		Prediction				
		*T*	*P*	*L*	*R*	*D*			*T*	*P*	*L*	*R*	*D*
**True**	T	0.9	0.04	0	0	0	**True**	T	0.8	0.1	0.03	0	0
	P	0.1	0.92	0.05	0	0		P	0.15	0.8	0.06	0	0
	L	0	0.04	0.9	0.02	0.02		L	0.05	0.1	0.82	0.1	0.05
	R	0	0	0.05	0.9	0.08		R	0	0	0.06	0.8	0.1
	D	0	0	0	0.08	0.9		D	0	0	0.03	0.1	0.85
Precision		$a_{11}$	$a_{12}$	$a_{13}$	$a_{14}$	$a_{15}$	Precision		$a_{21}$	$a_{22}$	$a_{23}$	$a_{24}$	$a_{25}$

**Table 3:** Tomato ripeness detection performance reported by five-fold cross-validation based on YOLOv3.

Average Performance of the Ripeness Detection for Flower-End Image Set				Average Performance of the Ripeness Detection for Stem-End Image Set			
	Precision	Recall	F1-Score		Precision	Recall	F1-Score
Turning	0.942	0.924	0.932	Turning	0.960	0.896	0.926
Pink	0.870	0.914	0.888	Pink	0.864	0.898	0.878
Light Red	0.920	0.920	0.918	Light Red	0.912	0.956	0.936
Red	0.980	0.972	0.978	Red	0.942	0.952	0.946
Deep Red	0.996	0.988	0.994	Deep Red	0.976	0.952	0.966
Average	0.942	0.944	0.942	Average	0.931	0.931	0.931

**Table 4:** Tomato ripeness detection performance reported by five-fold cross-validation based on two decision fusion methods: stochastic fusion versus equal-weighted fusion.

Equal Weight Decision Fusion $\left(\alpha =\frac{1}{M};\;M\;\mathrm{is}\;\mathrm{the}\;\mathrm{Number}\;\mathrm{of}\;\mathrm{Viewpoints}\right)$				Stochastic Decision Fusion			
	Precision	Recall	F1-Score		Precision	Recall	F1-Score
Turning	0.960	0.928	0.944	Turning	0.964	0.934	0.948
Pink	0.904	0.942	0.924	Pink	0.908	0.948	0.930
Light Red	0.962	0.962	0.964	Light Red	0.964	0.962	0.964
Red	0.986	0.984	0.988	Red	0.986	0.982	0.986
Deep Red	0.998	0.992	0.998	Deep Red	0.996	0.992	0.998
Average	0.962	0.962	0.964	Average	0.964	0.964	0.965

**Table 5:** Comparison of the detection performance of the SDF-ConvNets to other methods.

Model	Category	No. of Classes	No. of Images	Precision	Recall	F1-Score
Proposed (equal weighting)	Tomato	5	548	0.962	0.962	0.964
Proposed (stochastic weighting)	Tomato	5	548	0.964	0.964	0.965
YOLOv3 (w/o decision fusion)	Tomato	5	548	0.937	0.938	0.937
YOLOv3 [33]	Apple	2	878	0.908	0.922	0.915
ANN [17]	Tomato	3	768	-	-	0.902
SVM [32]	Tomato	2	82	0.976	0.988	0.982
LDA+SVM [16]	Tomato	5	250	-	-	0.908
Fuzzy classifier [15]	Tomato	6	36	0.952	0.967	0.953

## Data Availability

Not applicable.

## References

[1] Nagy A., Riczu P., Tamás J. (2016). Spectral evaluation of apple fruit ripening and pigment content alteration. Sci. Hortic..

[2] Dumas Y., Dadomo M., Di Lucca G., Grolier P. (2003). Effects of environmental factors and agricultural techniques on antioxidant content of tomatoes. J. Sci. Food Agric..

[3] Radzevičius A., Karklelienė R., Viskelis P., Bobinas C., Bobinaite R., Sakalauskiene S. (2009). Tomato (Lycopersicon esculentum Mill.) fruit quality and physiological parameters at different ripening stages of Lithuanian cultivars. Agron. Res..

[4] Gautier H., Diakou-Verdin V., Bénard C., Reich M., Buret M., Bourgaud F., Poëssel J.L., Caris-Veyrat C., Génard M. (2008). How Does Tomato Quality (Sugar, Acid, and Nutritional Quality) Vary with Ripening Stage, Temperature, and Irradiance?. J. Agric. Food Chem..

[5] Klee H.J., Giovannoni J.J. (2011). Genetics and Control of Tomato Fruit Ripening and Quality Attributes. Annu. Rev. Genet..

[6] Hoeberichts F.A., Der Plas L.H.W., Woltering E.J. (2002). Ethylene perception is required for the expression of tomato ripening-related genes and associated physiological changes even at advanced stages of ripening. Postharvest Biol. Technol..

[7] Barua S., Rahi T., Hossain O., Mazumder A., Sharmin R., Zaman T., Ghosh D., Ahmed S. (2018). Optimization of Ethylene inhibitor-mediated controlled ripening of tomato (Solanum lycopersicum L.). Adv. Agric. Sci..

[8] Jia W., Li Y., Qu R., Baranowski T., Burke L.E., Zhang H., Bai Y., Mancino J.M., Xu G., Mao Z.-H. (2019). Automatic food detection in egocentric images using artificial intelligence technology. Public Health Nutr..

[9] Ege T., Yanai K. (2018). Image-based food calorie estimation using recipe information. IEICE Trans. Inf. Syst..

[10] Kaur G., Kaushik A., Sharma S. (2019). Cooking is creating emotion: A study on hinglish sentiments of youtube cookery channels using semi-supervised approach. Big Data Cogn. Comput..

[11] Zhao Y., Gong L., Zhou B., Huang Y., Liu C. (2016). Detecting tomatoes in greenhouse scenes by combining AdaBoost classifier and colour analysis. Biosyst. Eng..

[12] Liu G., Mao S., Kim J.H. (2019). A mature-tomato detection algorithm using machine learning and color analysis. Sensors (Switzerland).

[13] Hu C., Liu X., Pan Z., Li P. (2019). Automatic Detection of Single Ripe Tomato on Plant Combining Faster R-CNN and Intuitionistic Fuzzy Set. IEEE Access.

[14] Sun J., He X., Wu M., Wu X., Shen J., Lu B. (2020). Detection of tomato organs based on convolutional neural network under the overlap and occlusion backgrounds. Mach. Vis. Appl..

[15] Goel N., Sehgal P. (2015). Fuzzy classification of pre-harvest tomatoes for ripeness estimation {\textendash} An approach based on automatic rule learning using decision tree. Appl. Soft Comput. J..

[16] El-Bendary N., El Hariri E., Hassanien A.E., Badr A. (2015). Using machine learning techniques for evaluating tomato ripeness. Expert Syst. Appl..

[17] Wan P., Toudeshki A., Tan H., Ehsani R. (2018). A methodology for fresh tomato maturity detection using computer vision. Comput. Electron. Agric..

[18] Nanni L., Ghidoni S., Brahnam S. (2017). Handcrafted vs. non-handcrafted features for computer vision classification. Pattern Recognit..

[19] Kamilaris A., Prenafeta-Boldú F.X. (2018). Deep learning in agriculture: A survey. Comput. Electron. Agric..

[20] Li C., Cao Q., Guo F. (2009). A method for color classification of fruits based on machine vision. WSEAS Trans. Syst..

[21] Arakeri M.P. (2016). Lakshmana Computer Vision Based Fruit Grading System for Quality Evaluation of Tomato in Agriculture industry. Procedia Comput. Sci..

[22] Simonyan K., Zisserman A., Ghahramani Z., Welling M., Cortes C., Lawrence N.D., Weinberger K.Q. (2014). Two-Stream Convolutional Networks for Action Recognition in Videos. Advances in Neural Information Processing Systems 27.

[23] Redmon J., Farhadi A. (2018). YOLOv3: An Incremental Improvement. pjreddie.com.

[24] Simonyan K., Zisserman A. (2014). Very Deep Convolutional Networks for Large-Scale Image Recognition. arXiv.

[25] Szegedy C., Liu W., Jia Y., Sermanet P., Reed S., Anguelov D., Erhan D., Vanhoucke V., Rabinovich A. (2014). Going Deeper with Convolutions. arXiv.

[26] Krizhevsky A., Sutskever I., Hinton G.E. (2012). Imagenet classification with deep convolutional neural networks. Adv. Neural Inf. Process..

[27] He K., Zhang X., Ren S., Sun J. Deep residual learning for image recognition. Proceedings of the IEEE Computer Society Conference on Computer Vision and Pattern Recognition.

[28] Ren S., He K., Girshick R., Sun J. (2017). Faster R-CNN: Towards real-time object detection with region proposal networks. IEEE Trans. Pattern Anal. Mach. Intell..

[29] Redmon J., Divvala S., Girshick R. You only look once: Unified, real-time object detection. Proceedings of the IEEE Conference on Computer Vision and Pattern Recognition.

[30] Yang Z., Yu Y., You C., Steinhardt J., Ma Y. (2020). Rethinking bias-variance trade-off for generalization of neural networks. arXiv.

[31] Redmon J. Darknet: Open Source Neural Networks in C. https://pjreddie.com/darknet/.

[32] Kumar S.D., Esakkirajan S., Bama S., Keerthiveena B. (2020). A Microcontroller based Machine Vision Approach for Tomato Grading and Sorting using SVM Classifier. Microprocess. Microsyst..

[33] Kuznetsova A., Maleva T., Soloviev V. Detecting Apples in Orchards Using YOLOv3 and YOLOv5 in General and Close-Up Images. Proceedings of the International Symposium on Neural Networks.

